# Identification of the estrogen receptor beta as a possible new tamoxifen-sensitive target in diffuse large B-cell lymphoma

**DOI:** 10.1038/s41408-022-00631-7

**Published:** 2022-03-07

**Authors:** Myra Langendonk, Mathilde R. W. de Jong, Nienke Smit, Jonas Seiler, Bart Reitsma, Emanuele Ammatuna, Andor W. J. M. Glaudemans, Anke van den Berg, Gerwin A. Huls, Lydia Visser, Tom van Meerten

**Affiliations:** 1 University of Groningen, University Medical Center Groningen, Department of Hematology, Groningen, the Netherlands; 2 University of Groningen, ERIBA, Genomic Instability in Development and Disease, Groningen, the Netherlands; 3 University of Groningen, University Medical center Groningen, Department of Nuclear Medicine and Molecular Imaging, Groningen, The Netherlands; 4 University of Groningen, University Medical Center Groningen, Department of Pathology and Medical Biology, Groningen, the Netherlands

**Keywords:** B-cell lymphoma, Targeted therapies, Cancer imaging

## Abstract

Diffuse large B-cell lymphoma (DLBCL) is the most common lymphoma subtype. Despite the proven efficacy of combined immunochemotherapy (R-CHOP) in the majority of patients, ~40% of DLBCL patients do not respond or will relapse and consequently have a very poor prognosis. The development of targeted therapies has not improved patient survival, underscoring the need for new treatment approaches. Using an unbiased genome-wide CD20 guilt-by-association approach in more than 1800 DLBCL patients, we previously identified the estrogen receptor beta (ERβ) as a new target in DLBCL. Here, we demonstrate that ERβ is expressed at significantly higher levels in DLBCL compared to normal B cells, and ERβ plays a role in the protection against apoptosis in DLBCL. Targeting of the ERβ with the selective estrogen receptor modulator tamoxifen reduces cell viability in all tested DLBCL cell lines. Tamoxifen-induced cell death was significantly decreased in an ERβ knock-out cell line. The activity of tamoxifen was confirmed in a xenograft human lymphoma model, as tumor growth decreased, and survival significantly improved. Finally, tamoxifen-treated breast cancer (BC) patients showed a significantly reduced risk of 38% for DLBCL compared to BC patients who did not receive tamoxifen. Our findings provide a rationale to investigate tamoxifen, a hormonal drug with a good safety profile, in DLBCL patients.

## Introduction

Diffuse large B-cell lymphoma (DLBCL) is the most common lymphoma subtype characterized by an aggressive clinical course. Standard immunochemotherapy with rituximab, cyclophosphamide, doxorubicin, vincristine, and prednisone (R-CHOP) cures approximately 60% of patients [1]. However, ~40% of DLBCL patients do not respond to standard therapy and consequently have a very poor prognosis [2]. Despite the development of numerous and often expensive (targeted/immuno) therapies over the last two decades, the survival of patients in this latter group has not improved, indicating the need for alternative treatment approaches [3].

We recently employed a CD20 guilt-by-association approach, using the gene expression profiles of 1804 DLBCL patients, to find novel treatment targets in DLBCL. To enable an efficient and accelerated clinical application of new drugs for DLBCL patients, we focused on targets for which clinically approved drugs were already available. Using these data we identified a potential novel target, the ESR2 gene, which codes for the estrogen receptor beta (ERβ) protein, a receptor that can be targeted with selective ER modulators (SERMs) such as tamoxifen [4].

Tamoxifen is one of the most widely used anticancer drugs, with over four decades of safe clinical use. Tamoxifen is used to treat patients with ERα-positive breast cancer (BC), either as an adjuvant therapy to reduce the risk of disease recurrence or as a therapy in patients with metastatic BC [5]. When bound by tamoxifen, ERα can no longer fulfill its normal function as a transcriptional activator of genes that stimulate cell growth and division. Furthermore, the binding of tamoxifen to ERα has been shown to induce apoptosis in BC cells [6]. Tamoxifen may also have an effect on ERβ, as ERβ expression has been associated with improved survival in tamoxifen-treated BC patients [7, 8]. In pre-clinical models for diseases such as BC, targeting of ERβ with tamoxifen or with a selective inhibitor of ERβ resulted in inhibition of tumor growth in xenograft models [9, 10].

ERα and ERβ have similar structures and ligation with SERMs or endogenous estrogens leads to biological effects in both receptor types. However, these effects may differ between normal and malignant tissues [11]. To date, reports on the (pre)clinical impact of ERβ have been contradictory and of uncertain relevance with respect to the prognosis and outcome of hormonal therapy treatments [12–16]. DLBCL is traditionally thought of as an “ER negative” malignancy on the basis of immunohistochemical staining for ERα. However, even though there are some reports about the status of ERβ expression in DLBCL [14, 17], SERMs such as tamoxifen have not yet been examined in DLBCL.

We set out to investigate ERβ expression in DLBCL cell lines and primary DLBCL as well as the potential effect of tamoxifen in wild-type and ERβ knock-out DLBCL cell lines and a xenograft lymphoma model. Finally, we performed a nationwide, population-based study to assess the incidence of DLBCL in BC patients treated with tamoxifen, using data from the Dutch Cancer Registry.

## Materials and methods

### Cell culture

DLBCL cell lines OCILY3, U2932, SUDHL2, SUDHL4, and SC1 were cultured in RPMI1640 (Lonza BioWhittaker, Walkersville, MD, USA) with 10% fetal bovine serum (FBS; HyClone Thermo Scientific, Waltham, MA, USA), 1% penicillin–streptomycin and 1% glutamine (Lonza BioWhittaker). DLBCL cell lines SUDHL5, SUDHL6 and SUDHL10 were cultured in RPMI1640 with 20% FBS. Breast cancer cell line MCF7 was cultured in (DMEM (Lonza BioWhittaker) with 10% FBS. The identity of the cell lines was checked periodically by STR profiling and regularly tested for *mycoplasma*. Before experiments, cells were cultured for a minimum of 24 h in phenol red-free RPMI1640 (Gibco, Waltham, MA, USA) supplemented with charcoal-stripped FBS (Sigma-Aldrich, St. Louis, MO, USA).

### Nanostring

A nCounter Custom Codeset consisting of capture and reporter probes was hybridized to 100 ng of RNA for 16 h at 65 °C. After hybridization, samples were loaded on a nCounter SPRINT Cartridge and processed on the nCounter SPRINT™ Profiler.

Expression data were analyzed using Nanostring’s nSolver analysis software (version 3.0.). Registered sample counts passing the standard QC parameters were processed: For technical variability normalization against internal positive controls was used, next normalization against housekeeping genes GAPDH, POLR2A, and WDR55 was performed.

### Western blot

Cells were lysed in RIPA buffer (50 mM Tris/150 mM NaCl/ 2.5 mM Na_2_EDTA/ 1% Triton X-100/ 0.5% sodium deoxycholate/0.1% SDS in dH_2_0) with 1% phenylmethanesulphonyl fluoride for 30-45 min on ice. Protein concentration was determined using the Pierce™ BCA Protein Assay Kit (Thermo Scientific). Electrophoresis and blotting were carried out according to standard protocols. Staining was performed with antibodies for ERβ (1:1000; PPZ0506, Invitrogen, Waltham, MA, USA), ERα (1:1000; Abcam, Cambridge, UK), PARP1 (1:1000; Cell Signaling Technology, Danvers, MA, USA), anti-phospho-Histone H2AX (Ser139) (1:1000, clone JBW301, Merck Millipore, Burlington, MA, USA), active caspase-3 (1:500; Cell Signaling Technology) and GAPDH (1:10.000; Novus Bio, Abingdon, UK) overnight at 4 °C in 5% milk.

### Immunohistochemistry

IHC was performed on FFPE tissue according to standard protocols, with appropriate positive and negative controls. FFPE tissue of randomly selected DLBCL cases was used. Surgical rest material falling under the “code for proper use” was used and was approved under RR202100080 (Local Testing Committee Pathology). Sections were stained with anti-ERβ (CWK-F12, 1:160, Developmental Studies Hybridoma Bank, Iowa, USA) after antigen retrieval with 10 mM TRIS/1 mM EDTA pH9 for 15 min at 120 °C, for an hour at room temperature.

### Metabolic activity (Resazurin) assay

Cells were incubated with increasing concentrations of tamoxifen (S1238, Selleckchem, Houston, TX, USA), Endoxifen (Selleckchem), PHTPP (Abcam) and diarylpropionitrile (DPN; Sigma-Aldrich) for 48 h. Cells were incubated with increasing concentrations of CHOP chemotherapy in combination with tamoxifen for 72 hours. CHOP was composed of cyclophosphamide (University Medical Center Groningen (UMCG) pharmacy), doxorubicin (Selleckchem), vincristine (UMCG pharmacy), and prednisone (Selleckchem), in a composition set at the clinical ratio of 83/5.5/0.16/11.1, respectively [18]. AlamarBlue (Thermo Fisher Scientific) was added 8 h prior to read-out (extinction 560 nm, emission 590 nm).

### Flow cytometry

PI staining for dead/alive (Sigma-Aldrich) and Annexin V staining for apoptosis (IQP-120F, IQ products, Groningen, NL) was performed by flow cytometry (FACSCalibur, BD Biosciences, Franklin Lakes NJ, USA). Rescue experiments were performed using 20 µM QVD-OPh hydrate (QVD; SML0063, Sigma-Aldrich) added 1 h before tamoxifen. Competition assays were performed using DPN, which was added 1 h before tamoxifen and incubated for 48 h at 37 °C.

### CRISPR/Cas9 mediated knockout

Custom crRNAs (guide sequences see Supplemental Table 1), tracrRNA, and recombinant Cas9 protein were purchased from IDT (Alt-R^®^ CRISPR-Cas9 crRNA XT; Alt-R^®^ CRISPR-Cas9 tracrRNA, ATTO™ 550; Alt-R^®^ S.p. HiFi Cas9 Nuclease V3, Coralville, IA, USA).

Transfections were performed using the SF Cell Line 4D NucleofectorTM X Kit L (Lonza). crRNA:tracrRNA duplexes (80 µM stocks) were performed according to the supplier’s instructions. Cas9/gRNA RNPs formed by combining 124 pmol crRNA:tracrRNA duplexes and 65 pmol Cas9 protein were used per transfection reaction. Cells were resuspended in SF buffer with supplement and transfected with RNPs by electroporation using the program FF120 on a 4D NucleofectorTM device (Lonza). Electroporated cells were cultured for four days before clonal selection using semi-solid medium (MethoCult, Stemcell, Vancouver, Canada). Knockout was validated by genomic PCR analysis. PCRs were prepared using Invitrogen Taq-polymerase. Cycling conditions were as follows: primary denaturation step at 94 °C for 5 min followed by 35 cycles of PCR (94 °C for 30 s, 56 °C for 45 s, 72 °C for 45 s) and 72 °C for 7 min. Primer pairs were, forward: 5′-TTCCCACTCCTCTGAGGTTAATA-3′ and reverse: 5′-GGAGACTAAGGC ACGAGAATTG-3′. PCR amplicons were analyzed by agarose gel electrophoresis and Sanger sequencing.

### Xenograft lymphoma model

Six-week-old female NSG (NOD.Cg-Prkdcscid ll2rgtm1Wjl/SzJ) mice were purchased from the Centrale Dienst Proefdieren (CDP) breeding facility within the University Medical Center Groningen. Mouse experiments were performed in accordance with national and institutional guidelines, and all experiments were approved by the Institutional Animal Care and Use Committee of the University of Groningen (IACUC-RuG). Ten female NSG mice were injected subcutaneously with U2932 cells in matrigel (Basement Membrane Matrix, 354234, Corning, 50/50, 1 million cells) in each flank and randomly divided into two groups of five. The treatment group received a 50 mg 60-day release tamoxifen pellet (Innovative Research of America) on day zero, which has been shown to raise levels of tamoxifen in serum to 0.07 µM [19] and was effective in a xenograft mouse model [20]. Tumor size was measured with caliper up to three times a week.

### ^18^F-FES-PET/CT imaging

^18^F-fluoroestradiol positron emission tomography, combined with low dose computed tomography (^18^F-FES PET/CT) was performed in a patient with proven DLBCL and breast cancer to evaluate and differentiate ER expression in the BC and lymphoma lesions. ^18^F-FES PET/CT was performed according to existing guidelines of the European Association of Nuclear Medicine [21, 22].

### Statistical analysis

All experiments were performed in triplicate. Continuous data are presented as median and range, categorical variables as number and percentage, univariate comparisons were done using Fishers’s or paired *t* tests. A *P* value of <0.05 was considered significant. Survival analyses were performed using the log-rank method. Data were analyzed using GraphPad Prism (GraphPad Prism [version 7.0b]; GraphPad Software, La Jolla, CA).

## Results

### Estrogen receptor β is overexpressed in DLBCL compared to normal B cells

**Fig. 1:**
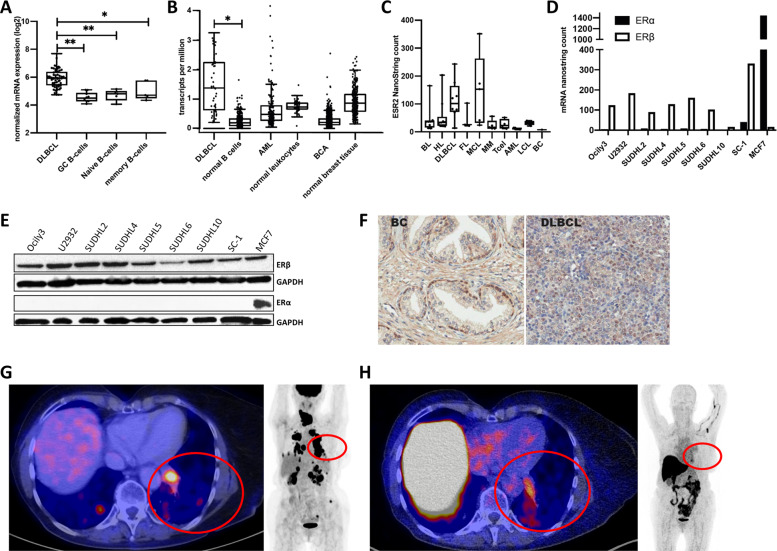
Estrogen receptor β (ERβ, gene: ESR2) is highly expressed in DLBCL. **A** Normalized ERβ mRNA expression levels for DLBCL cases and normal B-cell subsets (germinal center B cells, naive B cells, and memory B cells) obtained from a public repository (GSE12195). **B** ERβ RNA sequence expression data for DLBCL, normal B cells, acute myeloid leukemia (AML), normal leukocytes, breast cancer (BC), and normal breast tissue. **C** ERβ Nanostring counts for Burkitt lymphoma (BL), Hodgkin’s lymphoma (HL), DLBCL, follicular lymphoma (FL), mantle cell lymphoma (MCL), multiple myeloma (MM), T-cell lymphoma (T cell), AML, lymphoblastoïd cell lines (LCL), and BC cell lines. **D** ERα and ERβ Nanostring counts for DLBCL cell lines and MCF7. **E** Western blot of ERβ and ERα protein expression in DLBCL cell lines and the BC cell line MCF7. **F** Immunohistochemical staining for ERβ in normal breast and DLBCL patient tissue. **G** left fusion ^18^F-FDG PET/CT transaxial slide, right ^18^F-FDG PET Maximum Intensity Projection. **H** Left fusion ^18^F-FES PET/CT transaxial slide, right ^18^F-FES PET Maximum Intensity Projection.

We recently showed that ESR2 mRNA transcript levels are associated with the expression of CD20 mRNA levels in DLBCL and thus might serve as a target for treatment [4]. To determine the relevance of ERβ expression in DLBCL, we analyzed ESR2 mRNA expression in DLBCL patients and in normal B cells form healthy subjects using a publicly available gene expression data set (GSE12195, Supplemental Table 2). ESR2 mRNA expression was significantly higher in DLBCL than in normal B-cell subsets (germinal center B cells *P* < 0.001, naive B cells *P* < 0.001, and memory B cells *P* < 0.05, Fig. 1A). In addition, analysis of high-throughput RNA sequencing (RNA-Seq) data from the GEPIA database [23] showed that ESR2 expression is significantly higher in DLBCL cells than in normal B cells (*P* < 0.01). In contrast, ESR2 expression levels were not higher in other malignancies such as acute myeloblastic leukemia (AML) or BC compared to their normal counterparts (Fig. 1B). DLBCL appears to be one of the few tumor types in which ESR2 expression is significantly higher in malignant cells compared to their normal counterparts (Supplemental Fig. 1) [23]. Next, we determined ESR2 mRNA expression by NanoString in a cell line panel consisting of human B-cell lymphoma (*n* = 39), human T-cell lymphoma (*n* = 4), multiple myeloma (*n* = 5), AML (*N* = 5) and as a positive control the human BC cell line MCF7. MCF7 expressed both ESR2 and ESR1 [24]. ESR2 expression levels were highest in DLBCL and mantle cell lymphoma, followed by Burkitt lymphoma and Hodgkin lymphoma. The lowest ESR2 expression levels were found in the BC MCF7 cell line and in AML cell lines (Fig. 1C). In line with previous publications, no or only very low levels of ESR1 mRNA were detectable in DLBCL cell lines, whereas the BC MCF7 cell line showed high expression. By contrast, ESR2 mRNA levels were higher in DLBCL compared to BC (Fig. 1D).

ESR2 expression patterns in DLBCL patients were further characterized using a gene expression data set including 1,804 DLBCL patients (Supplemental Table 3). No differences in ESR2 expression levels were observed between male and female patients (Supplemental Fig. 2), nor in women aged <50 (mostly pre-menopausal) and >50 (mostly post-menopausal) (Supplemental Fig. 3). This suggests that ESR2 expression is not influenced by endogenous estrogen levels. Furthermore, ESR2 expression was significantly higher in germinal center subtype DLBCL patients than the activated B-cell subtype or unclassified DLBCL patients (*P* < 0.001) (Supplemental Fig. 4).

Next, we confirmed ERβ protein expression in DLBCL cell lines and primary DLBCL patient samples. In line with the mRNA data, we did not detect ERα protein expression in DLBCL cell lines, while ERα was highly expressed in the positive control BC cell line MCF7 (Fig. 1E). ERβ protein expression was detected in DLBCL and BC tumor cells using immunohistochemistry (IHC) (Fig. 1F). Of 91 DLBCL patients, 84 cases were positive, and 7 patients showed no staining. In a patient with DLBCL, we performed ^18^F-fluorodeoxyglucose positron emission tomography (^18^F-FDG PET/CT) imaging as standard for staging lymphoma. High ^18^F-FDG uptake was found in enlarged lymph nodes above the diaphragm, and in several lesions in the lungs, kidneys and bones (Fig. 1G). This patient also underwent ^18^F-fluoroestradiol (^18^F-FES) PET/CT imaging to evaluate ER expression. Increased ^18^F-FES uptake was found in intrapulmonary lesions, suggestive of increased ER receptor expression (Fig. 1H,). Based on all these data, we conclude that DLBCL is a malignancy with overexpression of ERβ and as such may be sensitive to ER-directed therapy.

### Tamoxifen is an ERβ antagonist in DLBCL

**Fig. 2:**
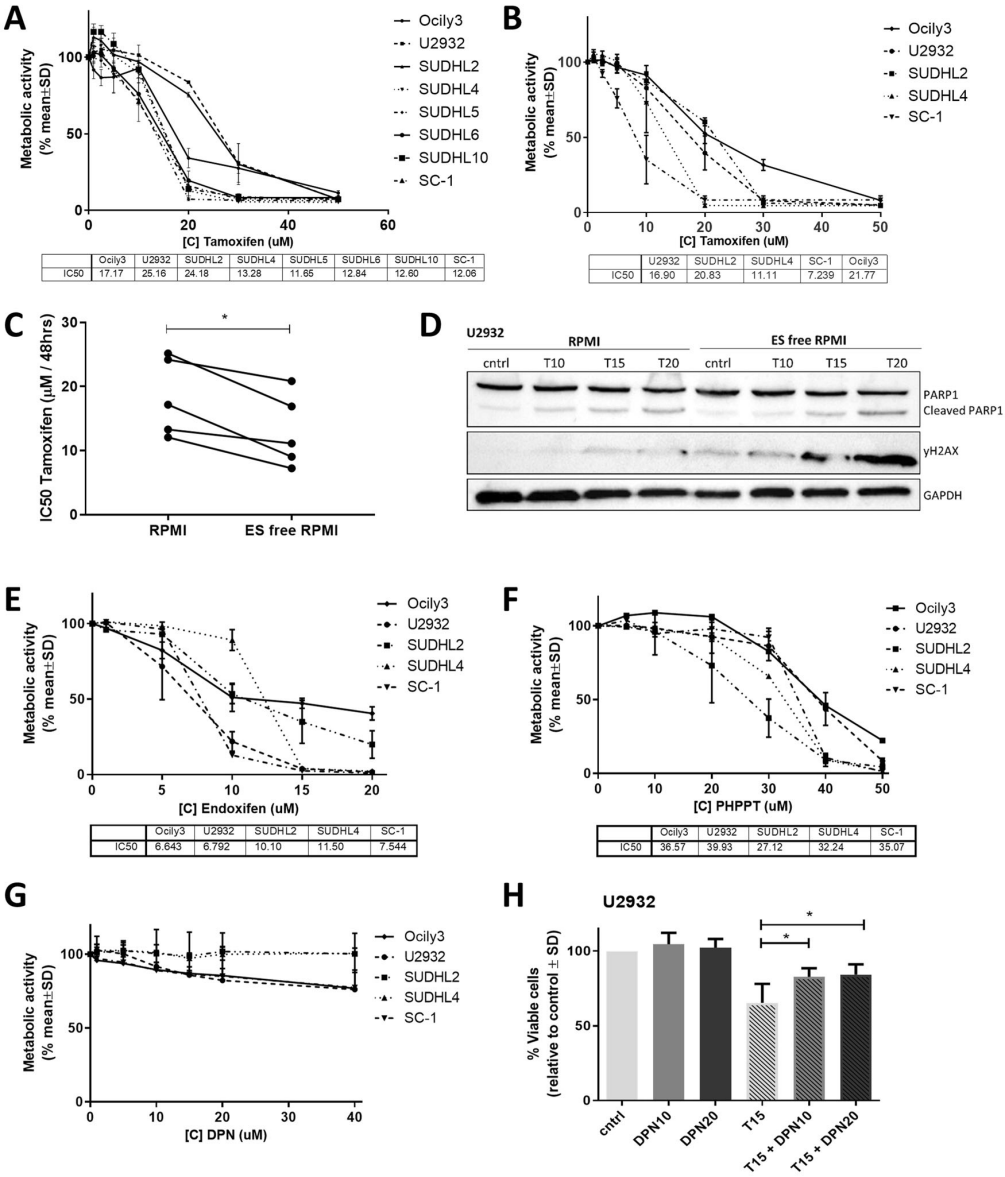
Tamoxifen is an ERβ antagonist in DLBCL. **A** Cell viability assay for DLBCL cell lines treated with tamoxifen (TAM) for 48 h in estrogen-containing culture medium. **B** Cell viability assay for cell lines treated with tamoxifen for 48 h in estrogen-free culture medium. **C** IC_50_ values for DLBCL cell lines treated with tamoxifen for 48 h on normal culture medium and ES-free culture medium. **D** Western blot of U2932 treated with 10 (T10), 15 (T15), and 20 (T20) µM of tamoxifen in RPMI or ES-free RPMI. The effect of Tamoxifen treatment is shown on apoptosis with PARP and DNA damage with γH2AX. **E** Cell viability assay for cell lines treated with endoxifen for 48 h. **F** Cell viability assay for cell lines treated with PHTPP for 48 h. **G** Cell viability assay for cell lines treated with DPN for 48 h. **H** Competition assay for U2932 treated with tamoxifen and DPN for 48 h. Data were normalized to the control and plotted as the mean ± standard deviation (SD) of *n* = 3 in all panels. *P ≤ 0.05.

The effects of SERMs, such as tamoxifen, on ERβ are diverse and complex since they can act as either antagonists or agonists [25]. We studied the in vitro effect of tamoxifen, including its active metabolite endoxifen, the specific ERβ antagonist (PHTPP), and the specific ERβ agonist (DPN) on DLBCL cell viability and growth. Tamoxifen treatment resulted in a clear dose-dependent effect on cell viability in all tested DLBCL cell lines (Fig. 2A). The half-maximal inhibitory concentrations (IC50s) of the DLBCL cell lines ranged from 11.6 µM in SUDHL5 cells to 25.2 µM in U2932. The effect of tamoxifen on viability was more pronounced in estrogen-free (ES-free) culture medium (Fig. 2B). The IC50 values decreased by 40% to 7.2 µM in SC1 and by 33% to 16.9 µM in U2932 cells. This decrease in IC50 was significant for the DLBCL cell line panel (Fig. 2C, *P* = 0.0125). In addition, we could show the effect on apoptosis and DNA damage by western blot on cell line U2932 (Fig. 2D). In ES-free medium at 20 µM tamoxifen more cleaved PARP is shown, and for 15 and 20 µM tamoxifen more γH2AX is found. In BC, it has been shown that estrogens can protect against apoptosis [26, 27]. As the effect of tamoxifen on cell viability was more pronounced in ES-free medium, this could be explained by competition between tamoxifen and ES or because of a protective effect against apoptosis. The effect of endoxifen on the viability of DLBCL cell lines was more pronounced than for tamoxifen, with IC50 values ranging from to 6.64 µM for OCILY3 cells to 11.5 µM for SUDHL4 (Fig. 2E). The selective ERβ antagonist PHTPP also decreased cell viability in all treated DLBCL cell lines, with SUDHL2 the most sensitive and U2932 the least sensitive (Fig. 2F). Although others have shown that the selective ERβ agonist DPN induces lymphoma cell death [17], we were not able to confirm this in our panel of DLBCL cell lines. Only two of our cell lines showed a trend toward decreased cell viability when exposed to high concentrations of DPN (Fig. 2G). To determine whether the effect of tamoxifen is ERβ specific, we simultaneously treated these cell lines with both DPN and tamoxifen for chemical competition. We found that the effect of tamoxifen was partially mitigated by the addition of DPN (Fig. 2H and Supplemental Fig. 5).

### Tamoxifen induces apoptosis in DLBCL and acts synergistically with CHOP chemotherapy

**Fig. 3:**
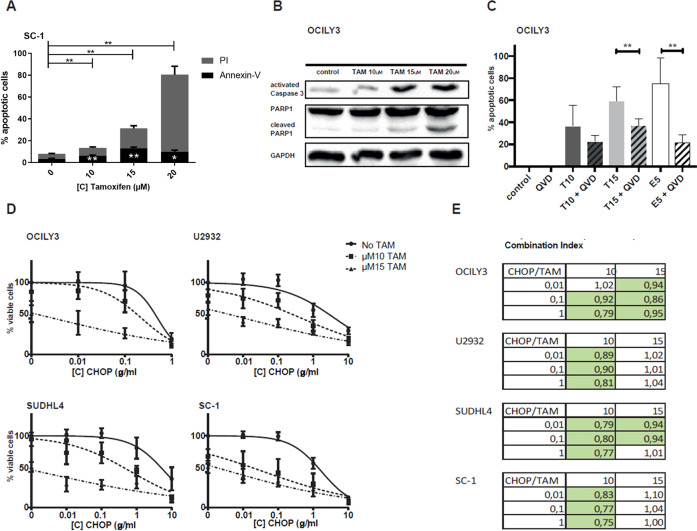
Tamoxifen induces apoptosis in DLBCL. **A** Annexin V/PI flow cytometry staining for apoptosis after 24 h tamoxifen treatment in SC1 cell line. **B** Western blot for PARP1/cleaved PARP1 and active caspase-3 after 24 h of tamoxifen treatment in OCILY3 cell line. **C** Rescue experiment for tamoxifen in OCILY3 cell line with caspase inhibitor QVD after 48 h treatment. Data were normalized to controls and are shown for three independent replicates. **D** Combination treatment of tamoxifen with CHOP chemotherapy for 72 h in four DLBCL cell lines. Data were normalized to controls and are shown for three independent replicates. **E** Combination index (CI) for the combination treatment of tamoxifen with CHOP chemotherapy for 72 h in four DLBCL cell lines. CI was calculated using CompuSyn software.

We then investigated whether the decreased viability of DLBCL cell lines after tamoxifen treatment was due to apoptosis. When the SC1 DLBCL cell line was treated with increasing doses of tamoxifen (10, 15, 20 µM) for 24 hours, the percentage annexin V/propidium iodide (PI) positive cells increased from 7.7% (control) to 13.5% (10 µM, *P* = 0.005), 31.7% (15 µM, *P* = 0.007), and 80.5% (20 µM, *P* = 0.003), respectively (Fig. 3A). Similar results were obtained with other cell lines (Supplemental Fig. 6). As already shown in Fig. 2D for U2932 that apoptosis is induced also for molecules that can be measured by western blot, treatment of OCILY3 cells with tamoxifen resulted in a dose-dependent increase in active caspase-3 and cleaved PARP1 (Fig. 3B). Addition of the pan-caspase inhibitor QVD rescued cells from tamoxifen-induced or etoposide-induced (positive control) apoptosis (Fig. 3C). This rescue was most robust when OCILY3 cells were treated with 15 µM tamoxifen in the presence of 20 µM QVD, with the percentage of viable cells increasing from 41 to 63% (*P* = 0.0083). Similar results were obtained for the other DLBCL cell lines (Supplemental Fig. 7). First-line CHOP chemotherapy is the backbone of the treatment for DLBCL. Here, we studied the combination of CHOP and tamoxifen treatment. As shown in Fig. 3D and E, the combination of CHOP with tamoxifen works well in DLBCL cell lines, and the effect of CHOP is more pronounced in ES-free medium (Supplemental fig. 8 and 9). The addition of 10 and 15 µM tamoxifen significantly reduces the cell viability where CHOP single treatment of 0.01 and 0.1 µg/mL has little to no effect on cell viability. The combination therapy of tamoxifen and CHOP is synergistic or additive in all cell lines shown with a combination index ranging from 1.1 (additive) to 0.75 (synergistic) [28].

### Knockout of ERβ reduces sensitivity to tamoxifen

**Fig. 4:**
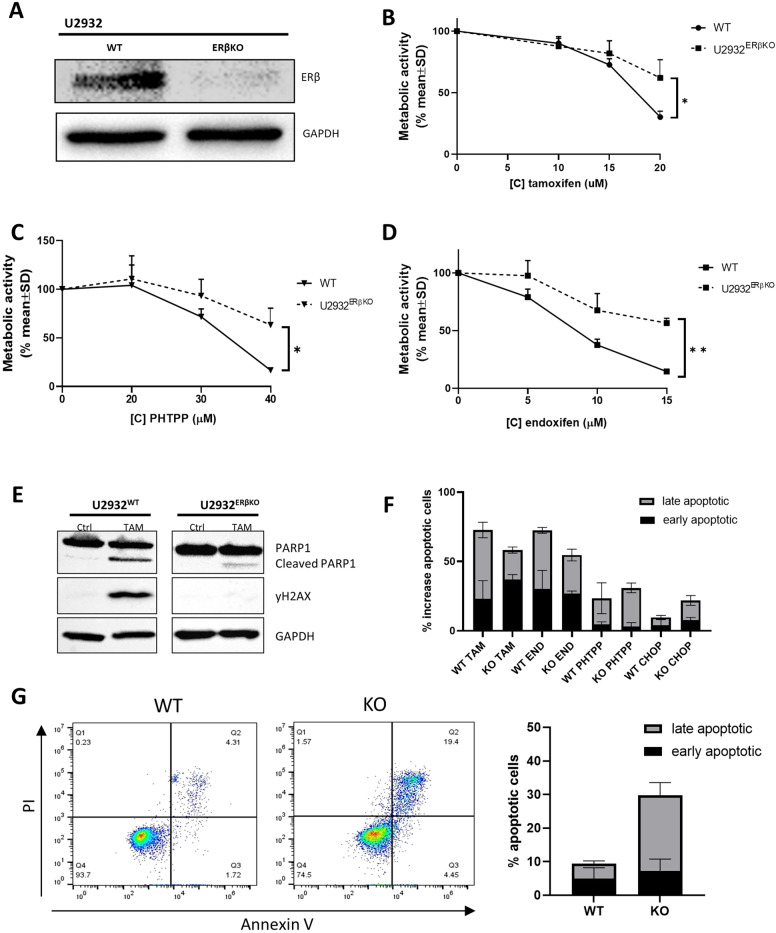
ERβ KO reduces sensitivity to tamoxifen, endoxifen, and selective ERβ antagonist PHTPP. **A** Western blot for ERβ in WT and KO cell line to confirm knockout. **B** Cell viability assay for WT and KO U2932 cell line treated with tamoxifen for 48 h. **C** Cell viability assay for WT and KO U2932 cell line treated with PHTPP for 48 h. **D** Cell viability assay for WT and KO U2932 cell line treated with endoxifen for 48 h. **E** Western blot for PARP1/cleaved PARP1 and γH2AX for U2932 and U2932 ^ERβO^ treated with 20 µM tamoxifen for 24 h. **F** Apoptosis induction with flow cytometry for AnV + (early) and AnV/PI + (late) apoptosis after treatment with tamoxifen (20 µM), endoxifen (15 µM), PHTPP (40 µM) and CHOP (1 µg/ml) in WT and ERβ KO U2932 cells. Average of three experiments. **G** Apoptosis for untreated WT and ERβ KO cells, flow cytometry for AnV and PI, and the average of four experiments.

To confirm that the effect of tamoxifen is mediated by its binding to ERβ, we generated an ERβ knock-out (KO) cell line. We confirmed loss of the ERβ gene by western blot, PCR, and sanger sequencing (Fig. 4A and supplemental Figs. 10 and11). Next, we treated the U2932^ERβKO^ and WT U2932 cell line with tamoxifen, PHTPP, and endoxifen (Fig. 4B–D). The KO resulted in a significantly decreased sensitivity to all three of the compounds, indicating that ERβ is indeed targeted by tamoxifen. We observed lower levels of cleaved PARP1 and γH2AX in U2932^ERβKO^ compared to WT U2932 (Fig. 4E), these results support that the binding of tamoxifen to ERβ is specific and causing the effect we see induced by tamoxifen in these cell lines. In the KO model, we tested apoptosis induction by tamoxifen, endoxifen and PHTPP. Total apoptosis induction, early (AnV positive), and late (AnV with PI positive) is lower in KO cells than WT although differences are small (Fig. 4F), probably because of the effect of ERβ KO on apoptosis protection. We therefore also tested the effect of CHOP on these cells and saw that the effect of CHOP was stronger on the KO cells than the WT (Fig. 4F). We next checked if KO cells without treatment showed more apoptosis than WT cells (30% in KO versus 10% in WT) (Fig. 4G). So although the effect of tamoxifen is specific for binding to ERβ, the loss of ERβ shows that the cells are protected from apoptosis by the expression of ERβ.

### Tamoxifen is effective in a mouse model of lymphoma

**Fig. 5:**
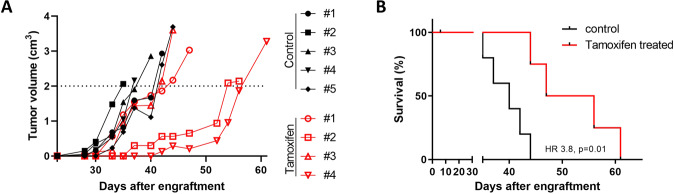
Tamoxifen is effective in a lymphoma mouse model. **A** Growth curve of tumor volume for individual mice (control in black and tamoxifen-treated mice in red). **B** Kaplan–Meier plot for the control group and tamoxifen-treated group. HR hazard ratio.

We explored the therapeutic potential of tamoxifen in vivo using a xenograft DLBCL mouse model. To obtain robust data, we used human DLBCL cell line U2932, one of the least tamoxifen-sensitive cell lines in vitro (Fig. 2A). One mouse in the treated group died on day 6 due to causes not related to tumor or treatment. Tumor outgrowth was achieved in all control mice and mice were sacrificed on days 35, 37, 40, 42, and 44. In the tamoxifen-treated mice tumor outgrowth was significantly slower in two of four mice which reached a tumor volume of >2 cm^3^ after 56 and 61 days (Fig. 5A), while the other two mice were sacrificed on days 44 and 47. Tamoxifen-treated mice also showed significantly improved overall survival (HR 3.8, *P* = 0.01) compared to control mice (Fig. 5B).

### Tamoxifen treatment results in a decreased incidence of DLBCL in breast cancer patients

**Fig. 6:**
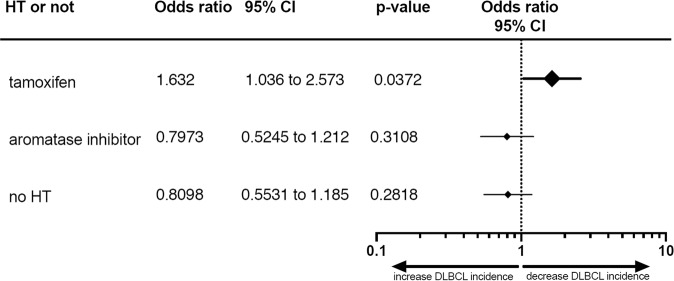
Tamoxifen decreases the incidence of DLBCL in BC patients. Odds ratio for patients treated with tamoxifen, aromatase inhibitors or no hormone therapy (HT), with 95% confidence intervals (CI) and *P* values.

To study the clinical relevance of tamoxifen for DLBCL, we performed a nationwide, population-based study to assess the incidence of DLBCL in BC patients treated with tamoxifen. Using the Dutch Cancer Registry, we selected all BC patients diagnosed in the Netherlands between 2007 and 2017 for whom follow-up was available. For clinical characteristics, see Supplemental Table 4. Over this ten-year period, 153,883 patients were diagnosed with BC. Around 83% of patients (*n* = 127,416) were ERα positive, of whom 68% received hormone therapy (HT) (tamoxifen or other HT). An additional 5.8% (*n* = 1452) of BC patients with an unknown or negative ER status also received HT. From a total cohort of 153,883 BC patients, 106 patients (0.07%) were diagnosed with DLBCL within 5 years after the start of BC treatment, of whom 24 were treated with tamoxifen, 31 were treated with an aromatase inhibitor, and 51 did not receive any HT. Apparently, in BC patients, DLBCL incidence was significantly reduced by tamoxifen treatment (Fisher exact test 0.0168, *P* < 0.05) compared to aromatase inhibitors or no HT, leading to a 38% reduction in relative risk for the development of DLBCL (Fig. 6 and Supplemental Table 5). As no effect was found for aromatase inhibitors on the incidence of DLBCL in BC patients, this additionally suggests that tamoxifen has a protective effect regarding DLBCL development.

## Discussion

Tamoxifen has been used as a hormonal therapy in a variety of cancers [29], but its efficacy has not yet been studied in DLBCL, the most common subtype of NHL. Our data presented in this manuscript show that ERβ, a tamoxifen target, is highly expressed in DLBCL, and that exposure of DLBCL cell lines to tamoxifen (either alone or in combination with CHOP chemotherapy) results in apoptosis and growth inhibition in vitro and in vivo. Overexpression of ER could also be visualized with ^18^F-FES PET/CT imaging, which might help in future selecting those patients that will have benefit from tamoxifen treatment. Furthermore, BC patients treated with tamoxifen show a reduced incidence of DLBCL. Together, these data suggest the potential importance of ERβ in the pathogenesis of DLBCL as well as a potential role for tamoxifen in the treatment of DLBCL.

In our patient cohort, 92% of patients showed expression of ERβ, this is more than the 53% that was previously reported [17]. They however used an antibody specific for ERβ1, while the antibody we used detects all isoforms of ERβ. They were also able to show a better prognosis for patients with negative or low expression in patients treated with R-CHOP, although the prognosis for the treatment with only CHOP was better for patients with Intermediate and high ERβ1 expression [17].

We showed that the effect of tamoxifen is caused by the binding to ERβ in different ways. We can show a similar effect with a specific ERβ antagonist (PHTPP), indicating the effect is specific for ERβ binding. We can also block the effect by pretreatment with DPN, a specific ERβ agonist, that in itself does not have an effect. As a third method we knocked out ERβ and that also decreased the effect of tamoxifen.

We found that estrogens can provide protection against apoptosis in DLBCL cell lines. This explains why it is beneficial for DLBCL cells to induce expression of ERβ in DLBCL compared to normal B cells. In the absence of estrogens, the IC50 for tamoxifen decreased, which could be due to competition between tamoxifen and estrogen for binding of the estrogen receptor. However, the increase in apoptosis in the KO cells suggests that estrogens also have an important role in the protection from apoptosis via ERβ. A protective effect has also been shown in ischemic heart disease, where upregulation of mitochondrial ERβ and the presence of estrogen had anti-apoptotic and cardio-protective effects [30, 31]. Moreover, in normal lung, kidney, bone marrow and BC cell lines overexpression of ERβ prevented cell apoptosis induced by different apoptotic stimuli [24]. The induction of apoptosis and DNA damage by tamoxifen could be due to the induction of reactive oxygen species (ROS), as has been reported in cell line MCF7 and that could be reversed by siRNA for ERβ but not ERα [32].

We used tamoxifen, endoxifen, and the ERβ antagonist PHTPP in our investigations, all of which induced apoptosis in DLBCL. Our approach differs from previous studies, which used specific ERβ agonists or estrogens to induce apoptosis while they did not use tamoxifen [17, 33–35]. One of the proposed mechanisms was a ligand-activated effect on lymphoma growth mediated by other cells in the micro-environment [33]. Direct effects on lymphoma cells have also been observed with different ERβ agonists [35]. However, these results were based on absolute cell counts after 96 h of ERβ agonist treatment and thus might reflect overstimulated or exhausted cells. In the majority of these studies, human and mice NHL cell lines other than DLBCL were used, which might also explain differences in results. We were not able to reproduce data regarding the treatment of NHL cells with ERβ agonists, such as DPN and a highly selective Erβ agonist KB9520 [35]; instead, we demonstrated that treatment with the clinically available SERM tamoxifen and the selective estrogen β antagonist PHTPP resulted in DLBCL cell death. Our findings are in line with results found in breast cancer by Ma et al., who showed a reduction in mammosphere formation after treatment with PHTPP and a reduction in patient-derived xenograft tumor volume after treatment with tamoxifen or PHTPP [9]. Although we realize that concentrations exceeding 10 μM are high, we demonstrated that a single administration of 10 μM tamoxifen induces apoptosis in the majority of DLBCL cell lines. Using the DLBCL cell line that was least tamoxifen-sensitive in vitro, we nevertheless demonstrated prominent effects of tamoxifen in a xenograft lymphoma mouse model. This result is especially remarkable, given that mice are unable to metabolize tamoxifen. The distribution of tamoxifen and its metabolites varies between serum and other tissues. The concentrations of tamoxifen and its metabolites in serum and tumor tissues significantly correlate, with five to ten times higher levels in tumor tissue compared to serum, reaching 1075 ng/ml (~3.5 µM) in tumor tissue with a daily dose of 20 mg tamoxifen [36]. Other data also suggest that the highest tamoxifen tissue concentrations are found in lymph nodes and cancer tissues [37]. Wide dose ranges of up to 200 mg/day are administered in clinical trials without apparent toxic effects [38, 39]. These findings confirm that reaching the therapeutic levels needed for effective treatment of DLBCL will be possible.

Analyzing a nationwide population-based cohort of BC patients, we showed that tamoxifen-treated BC patients have a lower incidence of DLBCL compared to those not treated with tamoxifen. The incidence and prognosis of DLBCL is generally worse in men than in women. Although DLBCL is not considered to be a hormone-related malignancy, epidemiological data on the gender difference suggest a causative role for estrogens [40]. Oral contraceptives and reproductive hormonal factors may reduce the risk of developing lymphoma [41]. On the other hand, data from the Women’s Health Initiative hormonal therapy trials, involving more than 25,000 women, did not demonstrate a clear correlation between estrogen and NHL (including DLBCL) development [42]. In this study, we used a different approach, examining DLBCL development in BC patients treated with tamoxifen. The observed risk reduction supports our pre-clinical data on the potential clinical value of tamoxifen in DLBCL. In addition, several anecdotal reports suggest an anticancer activity for tamoxifen in NHL or multiple myeloma patients [18, 43, 44]. The combination of tamoxifen with R-CHOP is a possible combination, another possibility would be the maintenance therapy with tamoxifen for patients with DLBCL that have a minimal residual disease or are high risk for relapse after R-CHOP.

In an era of rapid drug development, and of treatments associated with very high healthcare costs such as chimeric antigen receptor (CAR) T-cell cellular immunotherapy, we present data suggesting that tamoxifen, one of the most affordable and widely used anticancer therapies, might be a new treatment modality warranting further clinical evaluation in DLBCL.

## Supplementary information

supplemental material

## References

[1] Habermann TM, Weller EA, Morrison VA, Gascoyne RD, Cassileth PA, Cohn JB (2006). Rituximab-CHOP versus CHOP alone or with maintenance rituximab in older patients with diffuse large B-cell lymphoma. J Clin Oncol.

[2] Van Den Neste E, Schmitz N, Mounier N, Gill D, Linch D, Trneny M (2017). Outcomes of diffuse large B-cell lymphoma patients relapsing after autologous stem cell transplantation: an analysis of patients included in the CORAL study. Bone Marrow Transpl.

[3] Coiffier B, Sarkozy C (2016). Diffuse large B-cell lymphoma: R-CHOP failure-what to do?. Hematol Am Soc Hematol Educ Program.

[4] de Jong MRW, Visser L, Huls G, Diepstra A, van Vugt M, Ammatuna E (2018). Identification of relevant drugable targets in diffuse large B-cell lymphoma using a genome-wide unbiased CD20 guilt-by association approach. PLoS ONE.

[5] Tamoxifen for early breast cancer: an overview of the randomised trials. Early Breast Cancer Trialists’ Collaborative Group. Lancet. 1998;351:1451–67. 9605801

[6] Osborne CK (1998). Tamoxifen in the treatment of breast cancer. N. Engl J Med.

[7] Honma N, Horii R, Iwase T, Saji S, Younes M, Takubo K (2008). Clinical importance of estrogen receptor-beta evaluation in breast cancer patients treated with adjuvant tamoxifen therapy. J Clin Oncol.

[8] Gruvberger-Saal SK, Bendahl PO, Saal LH, Laakso M, Hegardt C, Eden P (2007). Estrogen receptor beta expression is associated with tamoxifen response in ERalpha-negative breast carcinoma. Clin Cancer Res.

[9] Ma R, Karthik GM, Lovrot J, Haglund F, Rosin G, Katchy A (2017). Estrogen receptor beta as a therapeutic target in breast cancer stem cells. J Natl Cancer Inst.

[10] Wu X, Subramaniam M, Grygo SB, Sun Z, Negron V, Lingle WL (2011). Estrogen receptor-beta sensitizes breast cancer cells to the anti-estrogenic actions of endoxifen. Breast Cancer Res.

[11] Gallo D, De Stefano I, Grazia Prisco M, Scambia G, Ferrandina G (2012). Estrogen receptor beta in cancer: an attractive target for therapy. Curr Pharm Des.

[12] Shaaban AM, Green AR, Karthik S, Alizadeh Y, Hughes TA, Harkins L (2008). Nuclear and cytoplasmic expression of ERbeta1, ERbeta2, and ERbeta5 identifies distinct prognostic outcome for breast cancer patients. Clin Cancer Res.

[13] Dey P, Jonsson P, Hartman J, Williams C, Strom A, Gustafsson JA (2012). Estrogen receptors beta1 and beta2 have opposing roles in regulating proliferation and bone metastasis genes in the prostate cancer cell line PC3. Mol Endocrinol.

[14] Faknuam S, Assanasen T, Ruangvejvorachai P, Hanvivadhanakul P, Intragumtornchai T, Rojnuckarin P (2018). Estrogen receptor beta expression and prognosis of diffuse large B cell lymphoma. Hematology.

[15] Huang B, Omoto Y, Iwase H, Yamashita H, Toyama T, Coombes RC (2014). Differential expression of estrogen receptor alpha, beta1, and beta2 in lobular and ductal breast cancer. Proc Natl Acad Sci USA.

[16] Ghali RM, Al-Mutawa MA, Al-Ansari AK, Zaied S, Bhiri H, Mahjoub T (2018). Differential association of ESR1 and ESR2 gene variants with the risk of breast cancer and associated features: a case-control study. Gene.

[17] Hasni MS, Berglund M, Yakimchuk K, Guan J, Linderoth J, Amini RM (2017). Estrogen receptor beta1 in diffuse large B-cell lymphoma growth and as a prognostic biomarker. Leuk Lymphoma.

[18] Decaudin D, Etienne MC, De Cremoux P, Maciorowski Z, Vantelon JM, Voog E (2004). Multicenter phase II feasibility trial of high-dose tamoxifen in patients with refractory or relapsed multiple myeloma. J Natl Cancer Inst.

[19] DeGregorio MW, Wilbur BJ, Coronado E, Osborne CK (1987). Serum tamoxifenconcentrations in the athymic nude mouse after three methods of administration. Cancer Chemother Pharm.

[20] Brosius SN, Turk AN, Byer SJ, Fromm Longo J, Kappes-JC, Roth KA (2014). Combinatorial therapy with tamoxifen and trifluoperazine effectively inhibits malignant peripheral nerve sheath tumor growth by targeting complementary signaling cascades. J Neuropathol Exp Neurol.

[21] Venema CM, Apollonio G, Hospers GA, Schröder CP, Dierckx RA, de Vries EF (2016). Recommendations and technical aspects of 16α-[18F]Fluoro-17β-estradiol PET to image the estrogen receptor in vivo: the Groningen experience. Clin Nucl Med.

[22] Glaudemans AWJM, de Vries EFJ. ^18^F FES PET/CT in oncology. Available at https://www.richtlijnendatabase.nl/gerelateerde_documenten/f/17259/18F%20FES%20PETCT%20in%20Oncology.pdf. Accessed 10/09, 2021.

[23] Tang Z, Li C, Kang B, Gao G, Li C, Zhang Z (2017). GEPIA: a web server for cancer and normal gene expression profiling and interactive analyses. Nucleic Acids Res.

[24] Liang J, Xie Q, Li P, Zhong X, Chen Y (2015). Mitochondrial estrogen receptor beta inhibits cell apoptosis via interaction with Bad in a ligand-independent manner. Mol Cell Biochem.

[25] Gallo MA, Kaufman D. Antagonistic and agonistic effects of tamoxifen: significance in human cancer. Semin Oncol. 1997;24(1 Suppl 1):S1-71–S1-80. 9045319

[26] Stanculescu A, Bembinster LA, Borgen K, Bergamaschi A, Wiley E, Frasor J (2010). Estrogen promotes breast cancer cell survival in an inhibitor of apoptosis (IAP)-dependent manner. Horm Cancer.

[27] Bailey ST, Shin H, Westerling T, Liu XS, Brown M (2012). Estrogen receptor prevents p53-dependent apoptosis in breast cancer. Proc Natl Acad Sci USA.

[28] Chou TC (2006). Theoretical basis, experimental design, and computerized simulation of synergism and antagonism in drug combination studies. Pharm Rev.

[29] Bogush TA, Polezhaev BB, Mamichev IA, Bogush EA, Polotsky BE, Tjulandin SA (2018). Tamoxifen never ceases to amaze: new findings on non-estrogen receptor molecular targets and mediated effects. Cancer Invest.

[30] Hsieh YC, Yu HP, Suzuki T, Choudhry MA, Schwacha MG, Bland KI (2006). Upregulation of mitochondrial respiratory complex IV by estrogen receptor-beta is critical for inhibiting mitochondrial apoptotic signaling and restoring cardiac functions following trauma-hemorrhage. J Mol Cell Cardiol.

[31] Schubert C, Raparelli V, Westphal C, Dworatzek E, Petrov G, Kararigas G, et al. Reduction of apoptosis and preservation of mitochondrial integrity under ischemia/reperfusion injury is mediated by estrogen receptor beta. Biol Sex Differ. 2016; 7:53-016–0104-8. 10.1186/s13293-016-0104-8 PMC5035458 27688871

[32] Razandi M, Pedram A, Jordan VC, Fuqua S, Levin ER (2013). Tamoxifen regulates cell fate through mitochondrial estrogen receptor beta in breast cancer. Oncogene.

[33] Yakimchuk K, Hasni MS, Guan J, Chao MP, Sander B, Okret S (2014). Inhibition of lymphoma vascularization and dissemination by estrogen receptor beta agonists. Blood.

[34] Pierdominici M, Maselli A, Locatelli SL, Ciarlo L, Careddu G, Patrizio M (2017). Estrogen receptor beta ligation inhibits Hodgkin lymphoma growth by inducing autophagy. Oncotarget.

[35] Yakimchuk K, Iravani M, Hasni MS, Rhonnstad P, Nilsson S, Jondal M (2011). Effect of ligand-activated estrogen receptor β on lymphoma growth in vitro and in vivo. Leukemia.

[36] Gjerde J, Gandini S, Guerrieri-Gonzaga A, Haugan Moi LL, Aristarco V, Mellgren G (2012). Tissue distribution of 4-hydroxy-N-desmethyltamoxifen and tamoxifen-N-oxide. Breast Cancer Res Treat.

[37] Furlanut M, Franceschi L, Pasqual E, Bacchetti S, Poz D, Giorda G (2007). Tamoxifen and its main metabolites serum and tissue concentrations in breast cancer women. Ther Drug Monit.

[38] Chamberlain MC, Kormanik PA (1999). Salvage chemotherapy with tamoxifen for recurrent anaplastic astrocytomas. Arch Neurol.

[39] Couldwell WT, Hinton DR, Surnock AA, DeGiorgio CM, Weiner LP, Apuzzo ML (1996). Treatment of recurrent malignant gliomas with chronic oral high-dose tamoxifen. Clin Cancer Res.

[40] Yildirim M, Kaya V, Demirpence O, Paydas S (2015). The role of gender in patients with diffuse large B cell lymphoma treated with rituximab-containing regimens: a meta-analysis. Arch Med Sci.

[41] Chihara D, Nastoupil LJ, Williams JN, Lee P, Koff JL, Flowers CR (2015). New insights into the epidemiology of non-Hodgkin lymphoma and implications for therapy. Expert Rev Anticancer Ther.

[42] Kato I, Chlebowski RT, Hou L, Wactawski-Wende J, Ray RM, Abrams J (2016). Menopausal estrogen therapy and non-Hodgkin’s lymphoma: a post-hoc analysis of women’s health initiative randomized clinical trial. Int J Cancer.

[43] Sola B, Renoir JM (2006). Antiestrogenic therapies in solid cancers and multiple myeloma. Curr Mol Med.

[44] Millis RR, Bobrow LG, Rubens RD, Isaacson PG (1988). Histiocytic lymphoma of breast responds to tamoxifen. Br J Cancer.

