# Supplementary material for: Identification of the estrogen receptor beta as a possible new tamoxifen-sensitive target in diffuse large B-cell lymphoma
Source: Blood Cancer J. 2022 Mar 7;12(3):36. doi: 10.1038/s41408-022-00631-7 (PMC8901714; doi:10.1038/s41408-022-00631-7)
Supplement: Supplementary file 1 — supplemental material [file 41408_2022_631_MOESM1_ESM.docx]

Supplemental Table 1. Guide sequences

| Guide sequences | |
| --- | --- |
| gRNA ESR2 Exon2 CDS | GAAGGAGAATTAAGGCTAGA |
| gRNA ESR2 Exon2 UTR | TCAGCTGTTATCTCAAGACA |

Supplemental Table 2. Gene expression omnibus (GEO) accession number (GSM), including Pubmed identifier (PMID).

| GSM# | Type of cell | PMID |
| --- | --- | --- |
| 306875-306882 | Germinal center B-cell | [19412164](https://www.ncbi.nlm.nih.gov/pubmed/19412164%22%20%5Co%20%22Link%20to%20PubMed%20record) |
| 476258-476262 | Naïve B-cell |  |
| 476263-476267 | Memory B-cell |  |
| 306886-387580 | DLBCL |  |

Supplemental Table 3. GSE accession numbers. Series identifiers and GPL platform numbers that were used in this study, including corresponding references.

| Series_identifier | Number of included patient samples | Platform | References |
| --- | --- | --- | --- |
| GSE10172 | 36 | GP L96 | 18509088 23143595 |
| GSE10524 | 15 | GP L570 | Citation missing |
| GSE10846 | 420 | GP L570 | 19038878 21546504 |
| GSE11318 | 10 | GP L570 | 18765795 |
| GSE12195 | 73 | GP L570 | 22137796 21390126 21156281 19965633 19412164 |
| GSE12453 | 10 | GP L570 | 18794340 24376854 |
| GSE12630 | 2 | GP L96 | 19332734 |
| GSE19246 | 59 | GP L570 | 20688907 |
| GSE22470 | 270 | GP L96 | 21487109 22238326 23143595 |
| GSE23501 | 26 | GP L570 | 20610814 |
| GSE25639 | 26 | GP L570 | 22689981 |
| GSE31312 | 364 | GP L570 | 26324762 26248897 26111978 22437443 23982177 23775435 |
| GSE34171 | 164 | GP L570 | 22975378 2376004 |
| GSE38885 | 21 | GP L570 | 23489474 |
| GSE44164 | 32 | GP L96 | 24179151 |
| GSE44337 | 9 | GP L570 | Citation missing |
| GSE4475 | 220 | GP L96 | 16760442 23143595 |
| GSE48097/GSE48184 | 29 | GP L570 | 24030260 |
| GSE57611/GSE57612 | 11 | GP L96 | 25042405 |
| GSE9429 | 7 | GP L96 | 19373655 |

Supplemental Table 4. Netherlands Cancer Registry diagnostics information.

|  | Total (n) | Total % | Total % of women with double diagnosis BC + DLBCL |
| --- | --- | --- | --- |
| Total women with BC diagnosed between 2007-2017 | 153883 | 100% |  |
| Total women with Breast Cancer and DLBCL  (during or after treatment of breast cancer with tamoxifen) | 106 | 0.07% | 100% |
| BC + DLBCL, treated with TAM | 24 | 0.016% | 22.6% |
| BC + DLBCL, NOT treated with TAM | 82 | 0.053% | 77.4% |

Supplemental Table 5. Number of patients with or without tamoxifen treatment.

|  | BC | BC+DLBCL |
| --- | --- | --- |
| Tamoxifen | 49737 | 24 |
| No tamoxifen | 104146 | 82 |


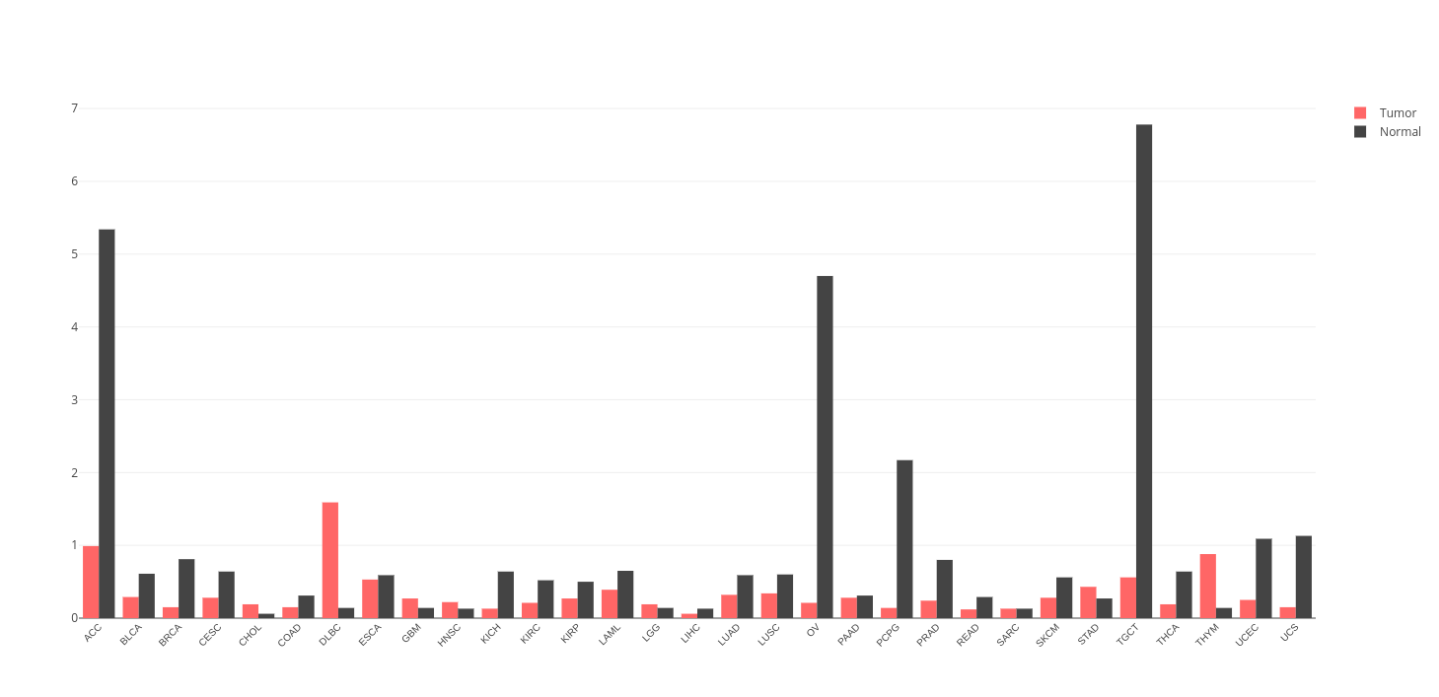
Supplemental Figure 1. RNA sequence data obtained from the online data base GEPIA. *ERβ* expression levels are shown. The black bars represent normal tissue and the red bars the malignant counterparts.

Abbreviations: ACC: Adrenocortical carcinoma, BLCA: Bladder Urothelial Carcinoma, BRCA: Breast invasive carcinoma, CESC: Cervical squamous cell carcinoma and endocervical adenocarcinoma, CHOL: Cholangio carcinoma, COAD: Colon adenocarcinoma, DLBC: Lymphoid Neoplasm Diffuse Large B-cell Lymphoma, ESCA: Esophageal carcinoma, GBM: Glioblastoma multiforme, HNSC: Head and Neck squamous cell carcinoma, KICH: Kidney Chromophobe, KIRC: Kidney renal clear cell carcinoma, KIRP: Kidney renal papillary cell carcinoma, LAML: Acute Myeloid Leukemia LGG: Brain Lower Grade Glioma, LIHC: Liver hepatocellular carcinoma, LUAD: Lung adenocarcinoma, LUSC: Lung squamous cell carcinoma, MESO: Mesothelioma, OV: Ovarian serous cystadenocarcinoma, PAAD: Pancreatic adenocarcinoma, PCPG: Pheochromocytoma and Paraganglioma, PRAD: Prostate adenocarcinoma, READ: Rectum adenocarcinoma, SARC: Sarcoma, SKCM: Skin Cutaneous Melanoma, STAD: Stomach adenocarcinoma, TGCT: Testicular Germ Cell Tumors, THCA: Thyroid carcinoma, THYM: Thymoma, UCEC: Uterine Corpus Endometrial Carcinoma, UCS: Uterine Carcinosarcoma, UVM: Uveal Melanoma


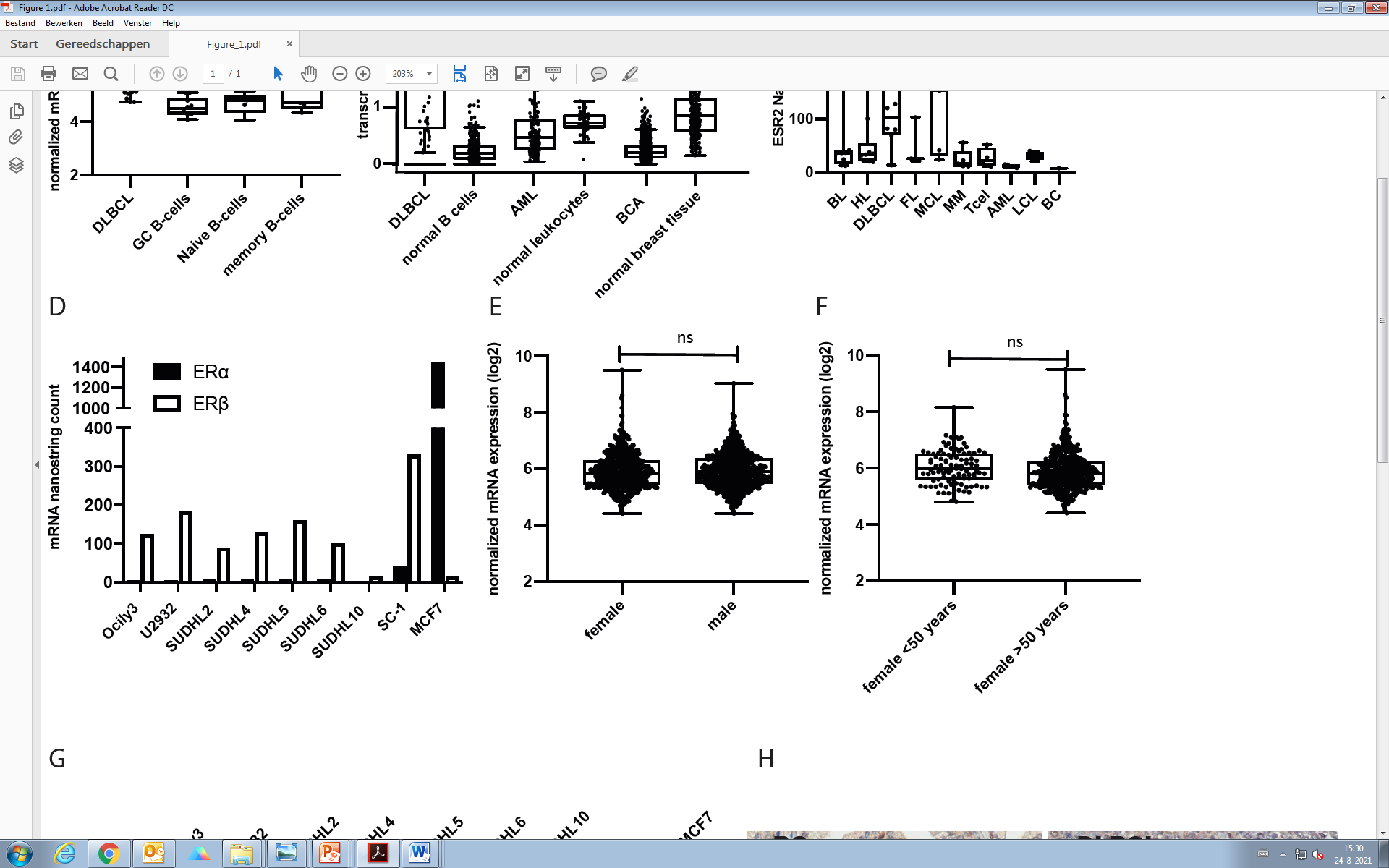


Supplemental Figure 2. Normalized ERβ mRNA expression levels in males and females.


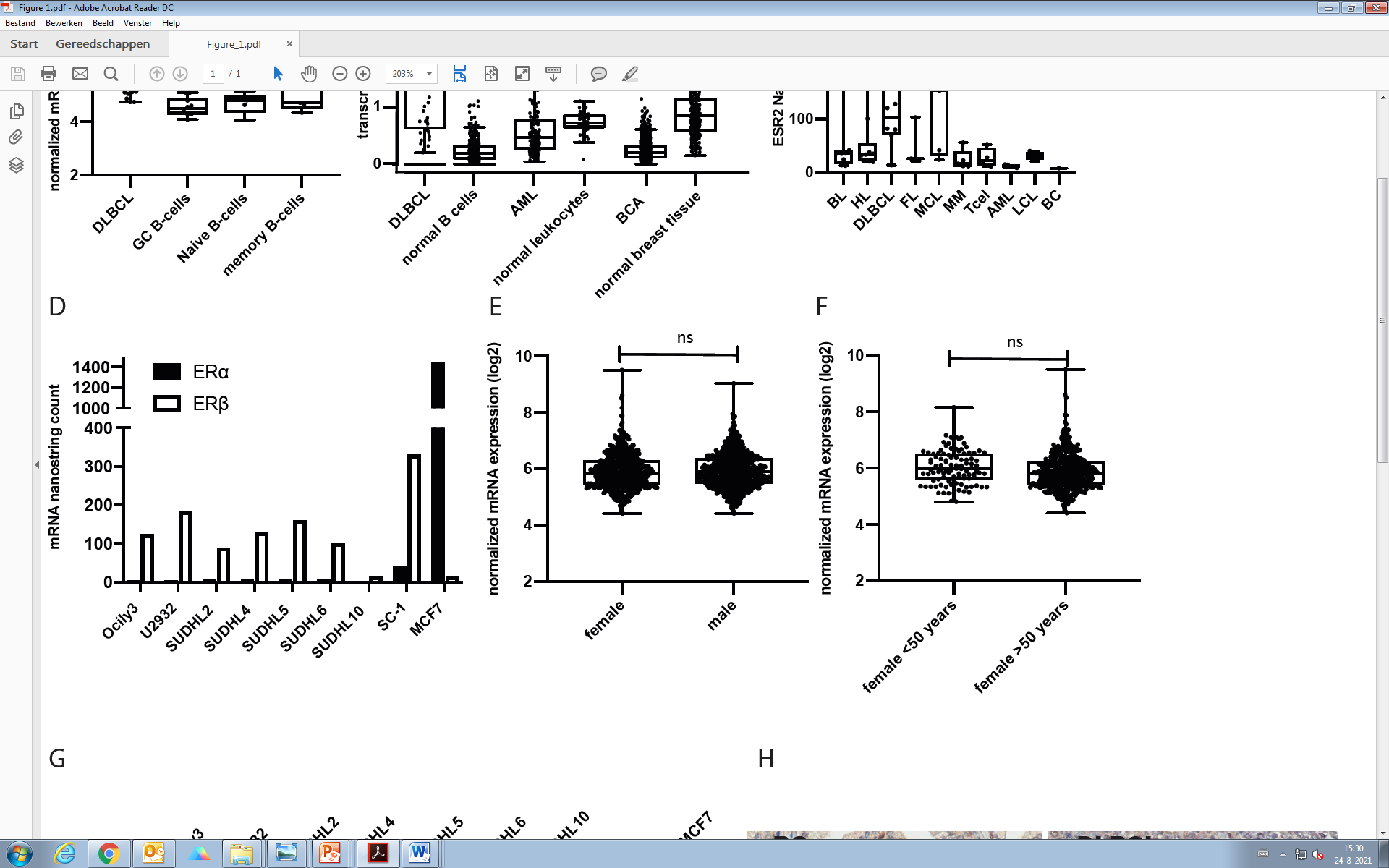


Supplemental Figure 3. Normalized ERβ mRNA expression levels in females above and below the age of 50 years.


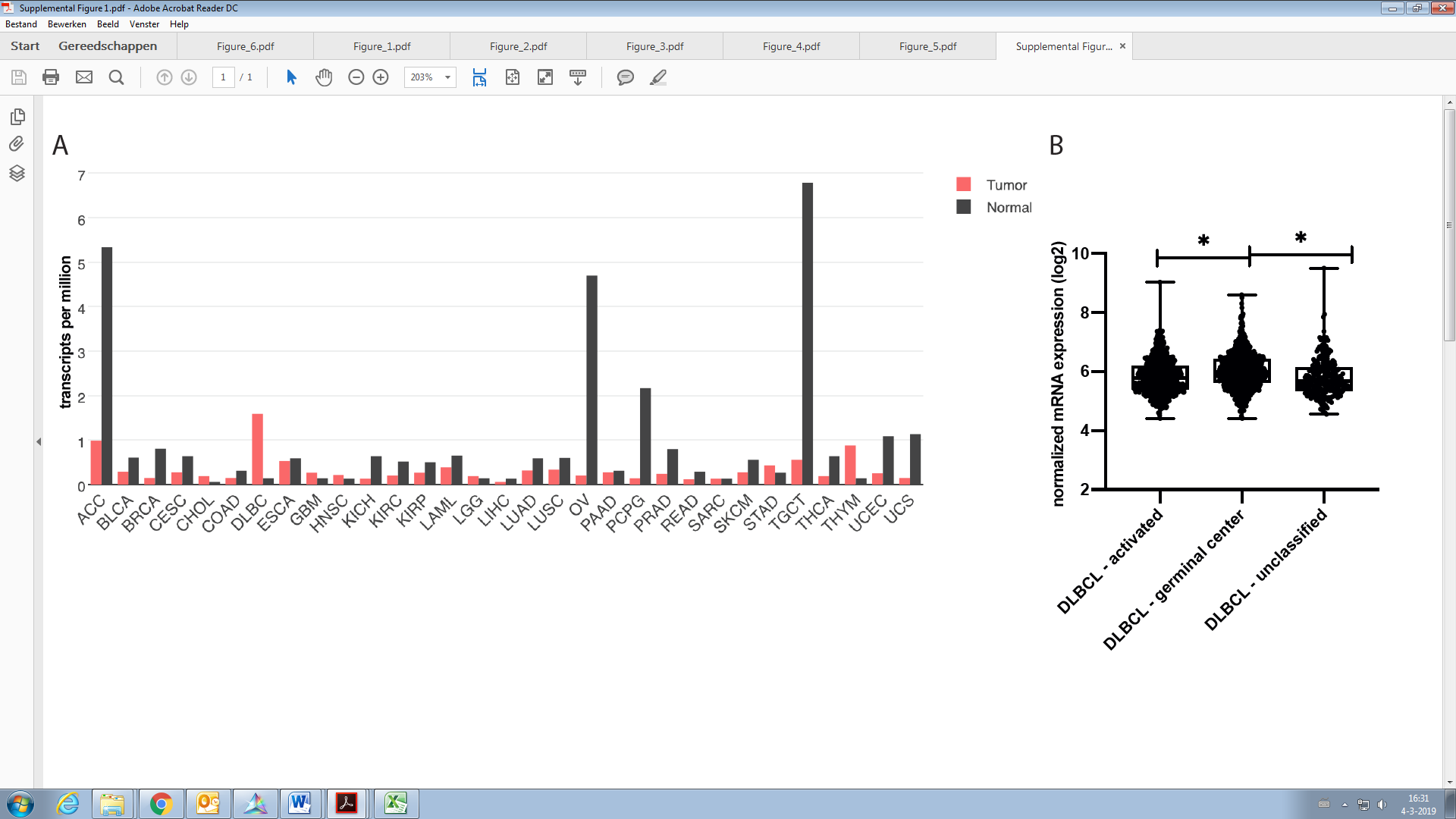


Supplemental Figure 4. ERβ mRNA expression is highest in GCB subtype compared to ABC subtype and unclassified subtype. * p ≤ 0.05


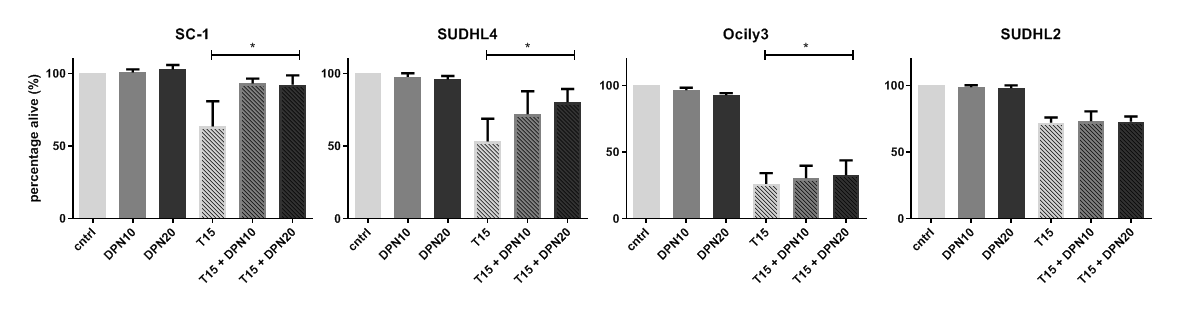


Supplemental figure 5. Competition assay for DLBCL cell lines SC-1, SUDHL4, OciLy3 and SUDHL2 treated with tamoxifen and DPN for 48h.


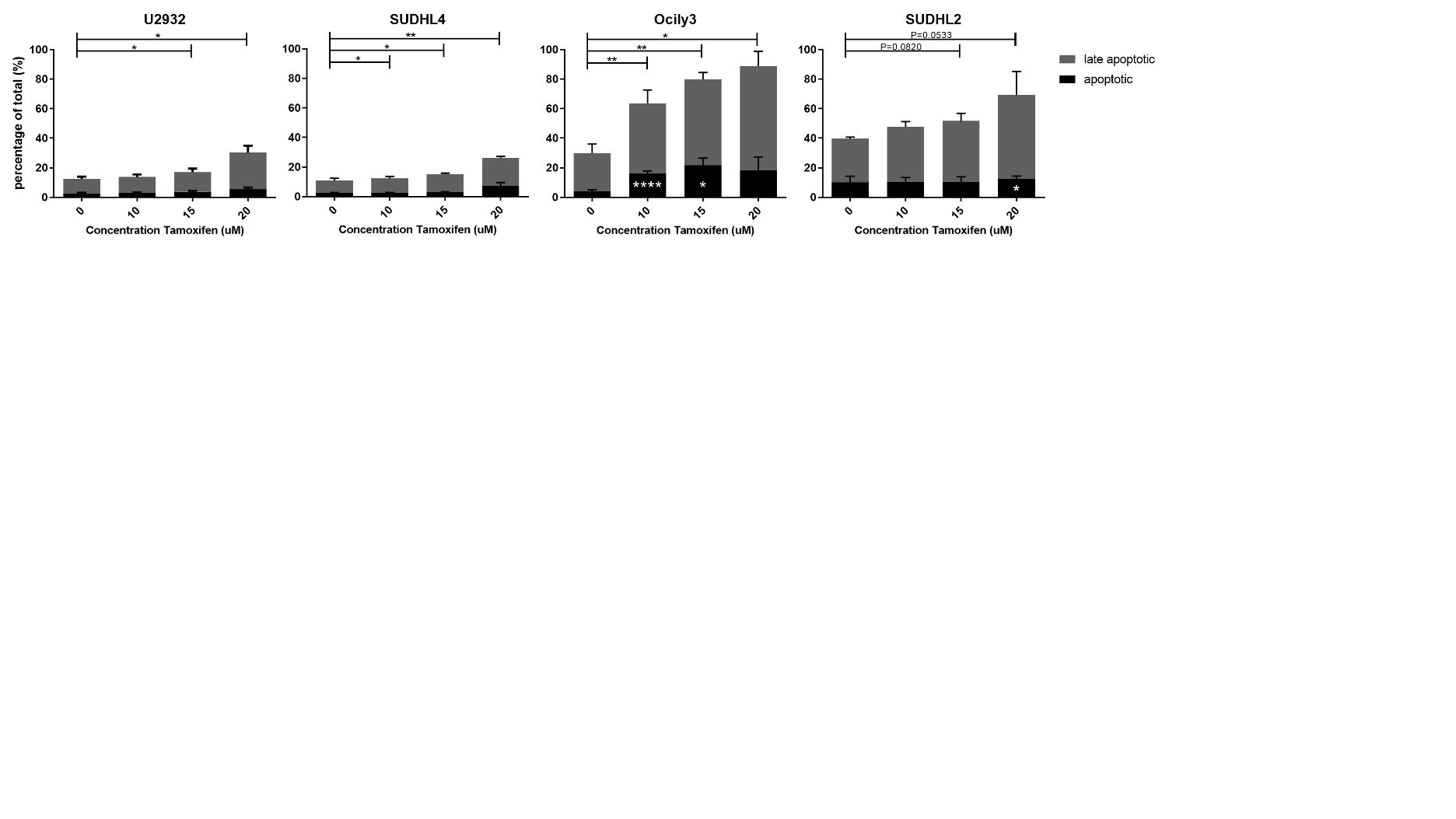


Supplemental Figure 6. Annexin V / PI flow cytometry staining for apoptosis after 24h tamoxifen treatment in DLBCL cell lines. Percentage early and late apoptotic cells for cell lines U2932, SC-1, SUDHL4 and SUDHL2. * p ≤ 0.05; ** p ≤ 0.01; **** p < 0.0001.


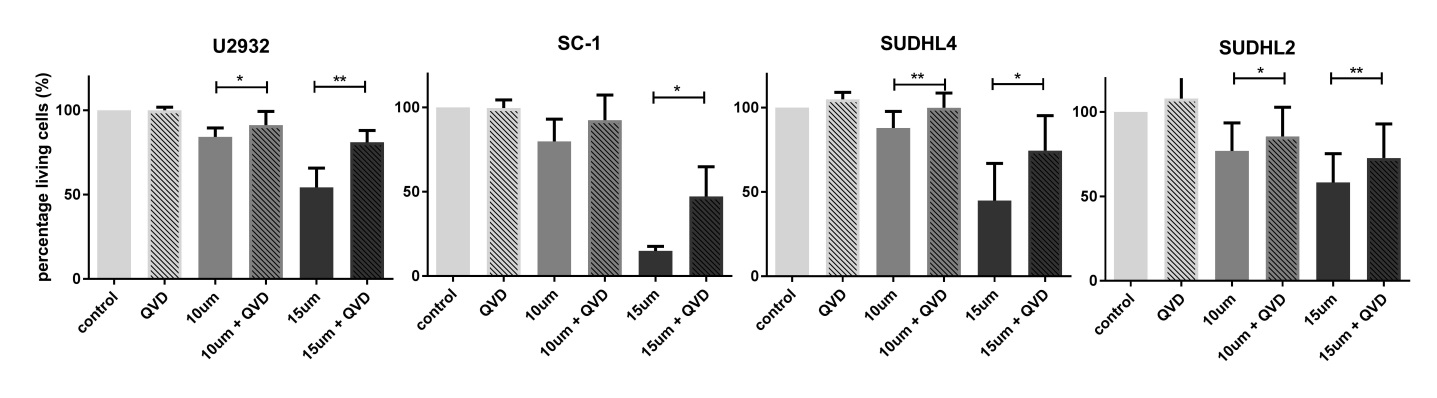


Supplemental Figure 7. QVD rescue experiments for tamoxifen in DLBCL cell lines U2932, SC-1, SUDHL4 and SUDHL2 after 48h. * p ≤ 0.05; ** p ≤ 0.01.


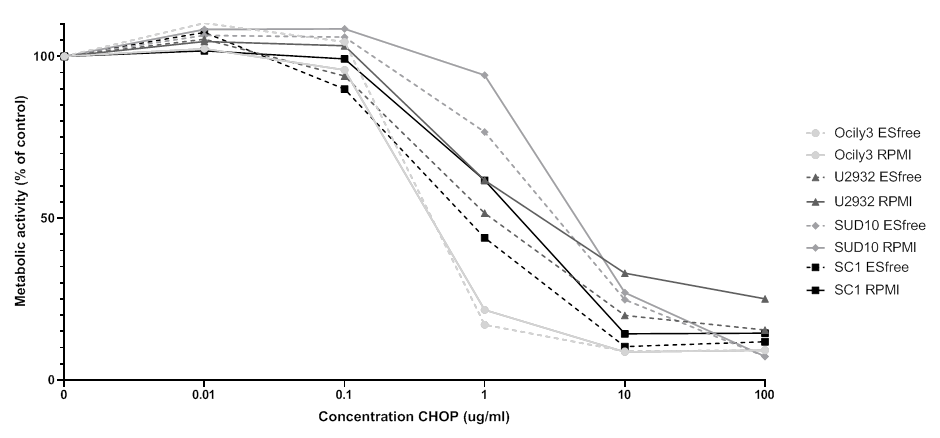


Supplemental Figure 8. Cell viability assay for cell lines treated with CHOP for 72h.

Supplemental Figure 9. Difference between IC50 values for cell lines treated with CHOP for 72h in normal RPMI and ES free RMPI.


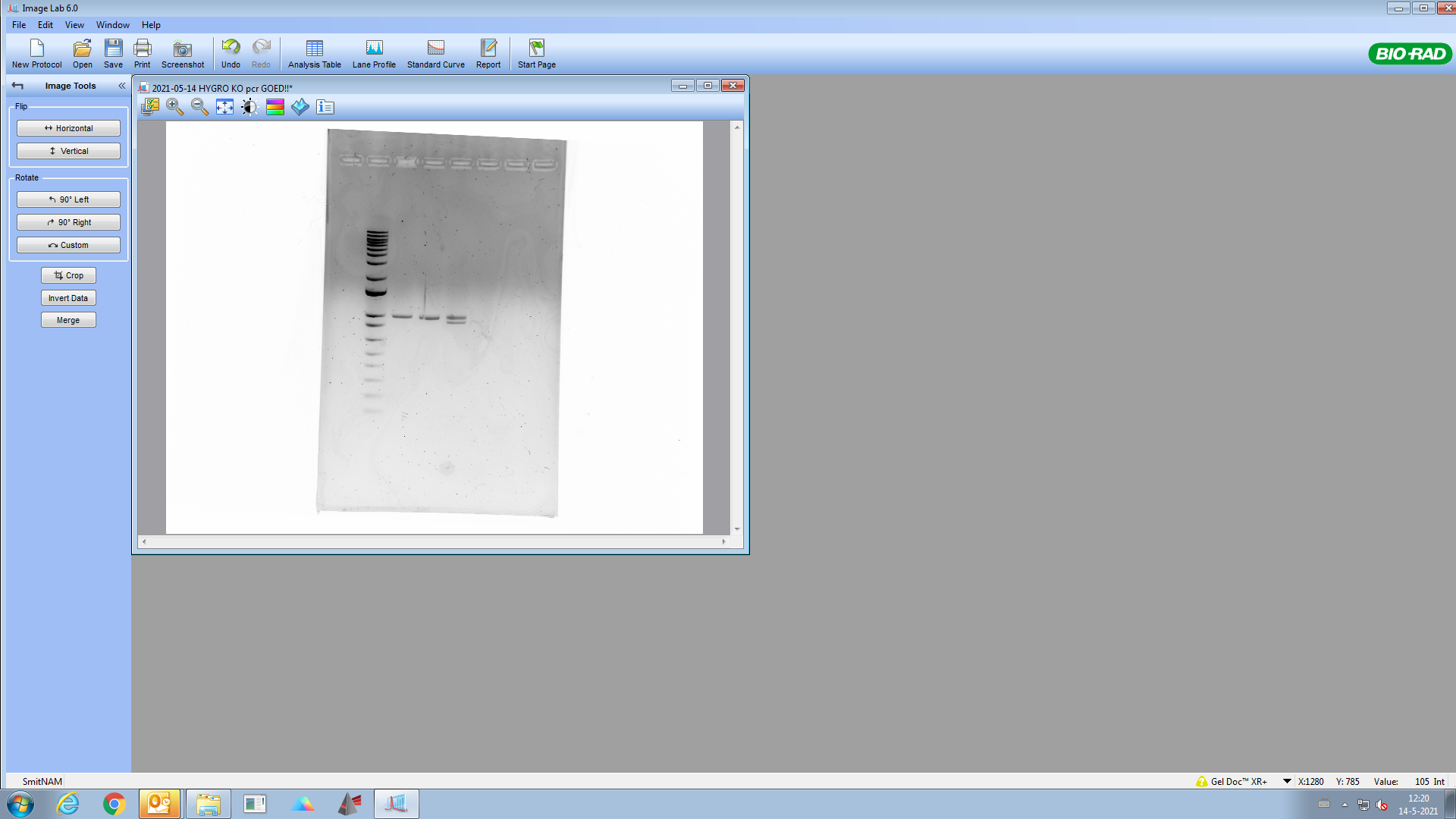


1kb ladder

WT

ERβKO

1000bp

850bp

Supplemental figure 10. PCR U2932 ERβKO


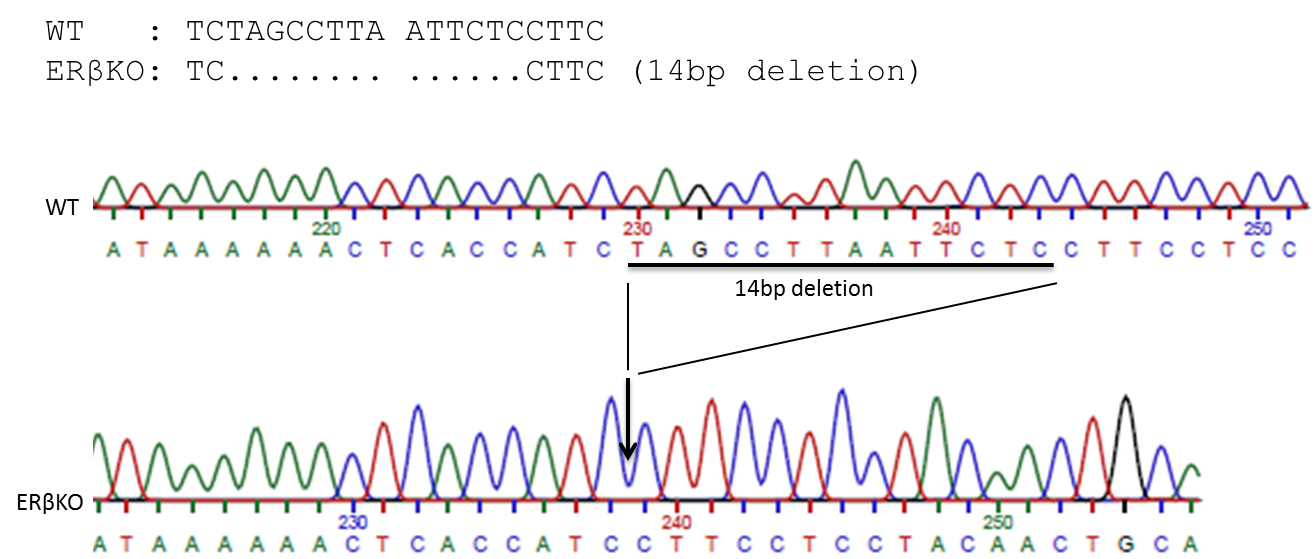


Supplemental figure 11. Sanger sequencing results U2932 WT and U2932 ERβKO
